# Erratum To: Production of recombinant *Entamoeba histolytica*pyruvate phosphate dikinase and its application in a lateral flow dipstick test for amoebic liver abscess

**DOI:** 10.1186/1471-2334-14-533

**Published:** 2014-11-10

**Authors:** Syazwan Saidin, Muhammad Hafiznur Yunus, Nor Dyana Zakaria, Khairunisak Abdul Razak, Lim Boon Huat, Nurulhasanah Othman, Rahmah Noordin

**Affiliations:** Institute for Research in Molecular Medicine, Universiti Sains Malaysia, Penang, 11800 Malaysia; School of Materials and Mineral Resources Engineering, Engineering Campus, Universiti Sains Malaysia, 14300 Nibong Tebal, Penang, Malaysia; NanoBiotechnology Research and Innovation (NanoBRI) Institute for Research in Molecular Medicine, Universiti Sains Malaysia, Penang, 11800 Malaysia; School of Health Sciences, Universiti Sains Malaysia, Kubang Kerian, Kelantan, 16150 Malaysia

## Correction

After publication of this article [1], it was brought to our attention that we had failed to make it clear that Figures one (Figure 1 here), two (Figure 2 here) and three (Figure 3 here) were put together by grouping elements from different images. The revised figures, in which this grouping has been made explicit in the arrangement, and figure legends, in which the adjustments are clearly stated, are shown below.

**Figure 1:**
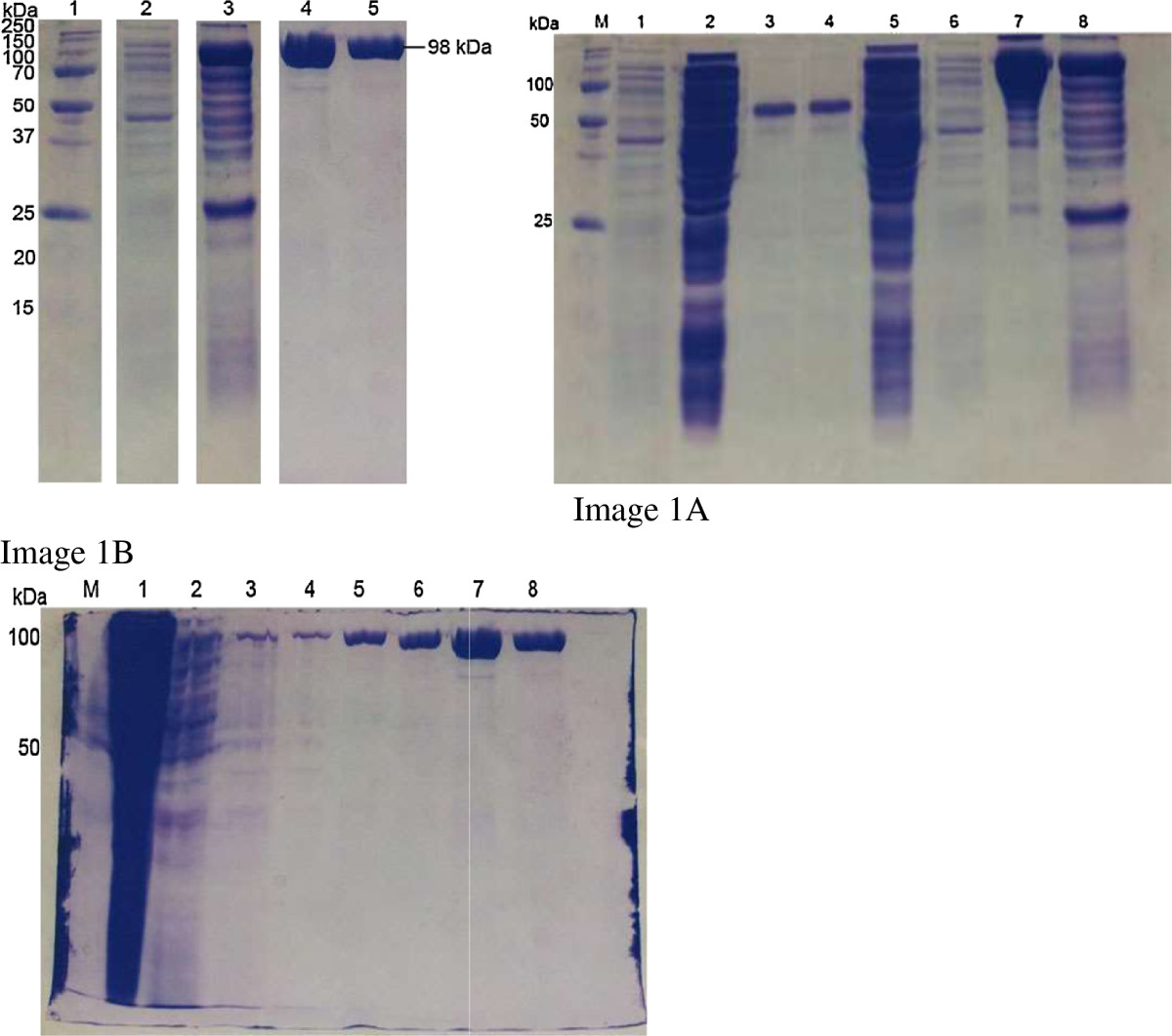
**SDS-PAGE analysis shows the purified rPPDK.** Lane 1: Precision Plus Protein™ Unstained Standard Marker (Bio-Rad, USA); Lane 2: Non-induced cell from pET28a/PPDK; Lane 3: Induced cell from pET28a/PPDK; Lanes 4–5: purified rPPDK protein from elution buffer (250 mM imidazole). Arrow indicates the recombinant PPDK protein (~98 kDa). Figure one (Figure 1 here) was made by grouping elements from the original Images 1**A** and 1**B** as follows: Lanes 1, 2 and 3 in Figure one (Figure 1 here) came from lanes M, 6 and 8 respectively in Image 1A; while Lanes 4 and 5 in Figure one (Figure 1 here) came from lanes 7 and 8 respectively in Image 1B.

**Figure 2:**
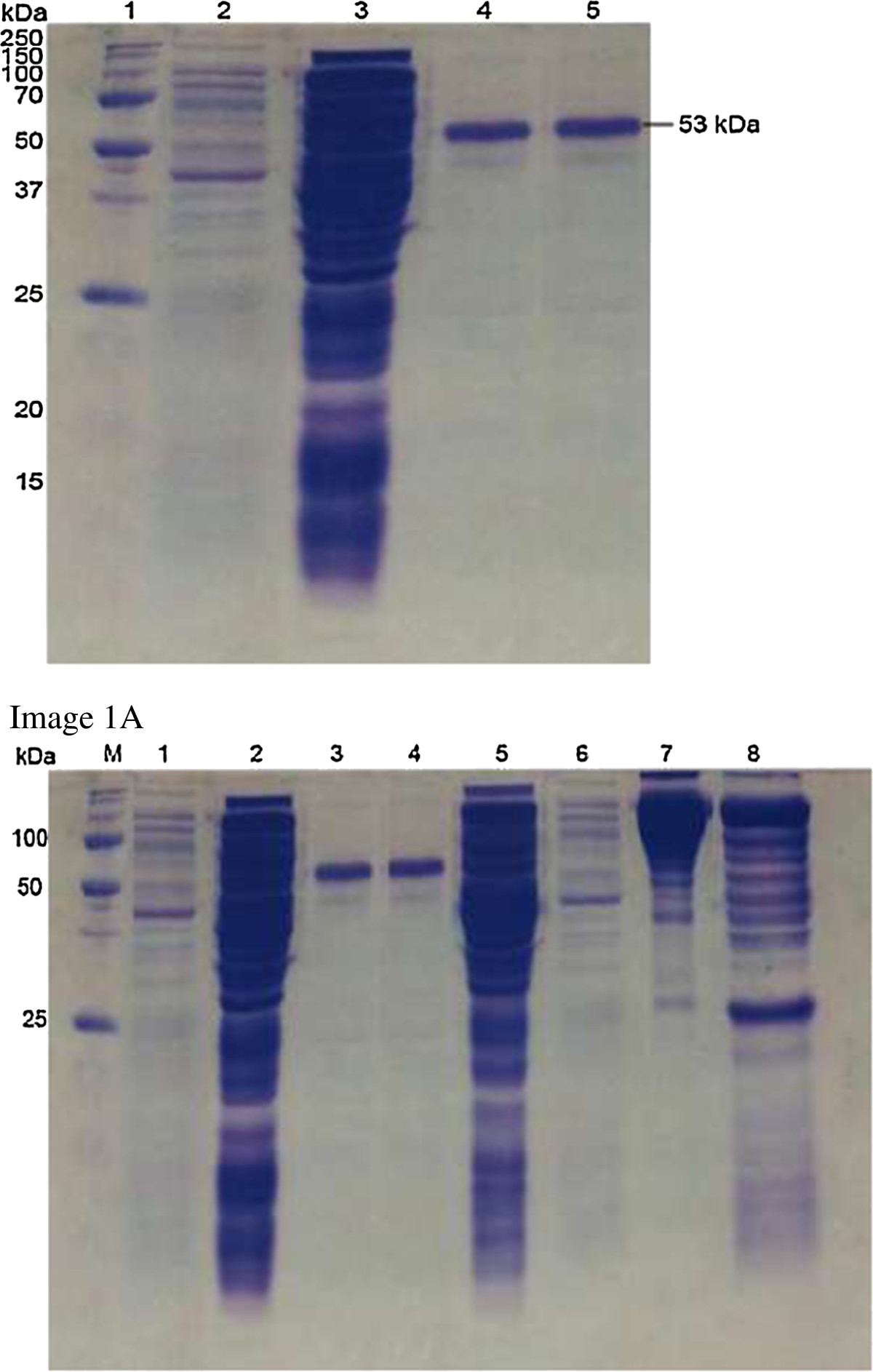
**SDS-PAGE analysis shows the purified rGal/GalNAc lectin.** Lane 1: Precision Plus Protein™ Unstained Standard Marker (Bio-Rad, USA); Lane 2: Non-induced cell from pET28a/Gal-GalNAc lectin; Lane 3: Induced cell from pET28a/Gal-GalNAc lectin; Lanes 4–5: purified rGal/GalNAc lectin protein from elution buffer (250 mM imidazole). Arrow indicates the recombinant Gal/GalNAc lectin protein (~53 kDa). Figure two (Figure 2 here) was made by grouping elements from the original image 1**A** as follows: Lanes 1, 2, 3, 4, and 5 in Figure two (Figure 2 here) came from lanes M, 1, 2, 3 and 4 respectively in Image 1A.

**Figure 3:**
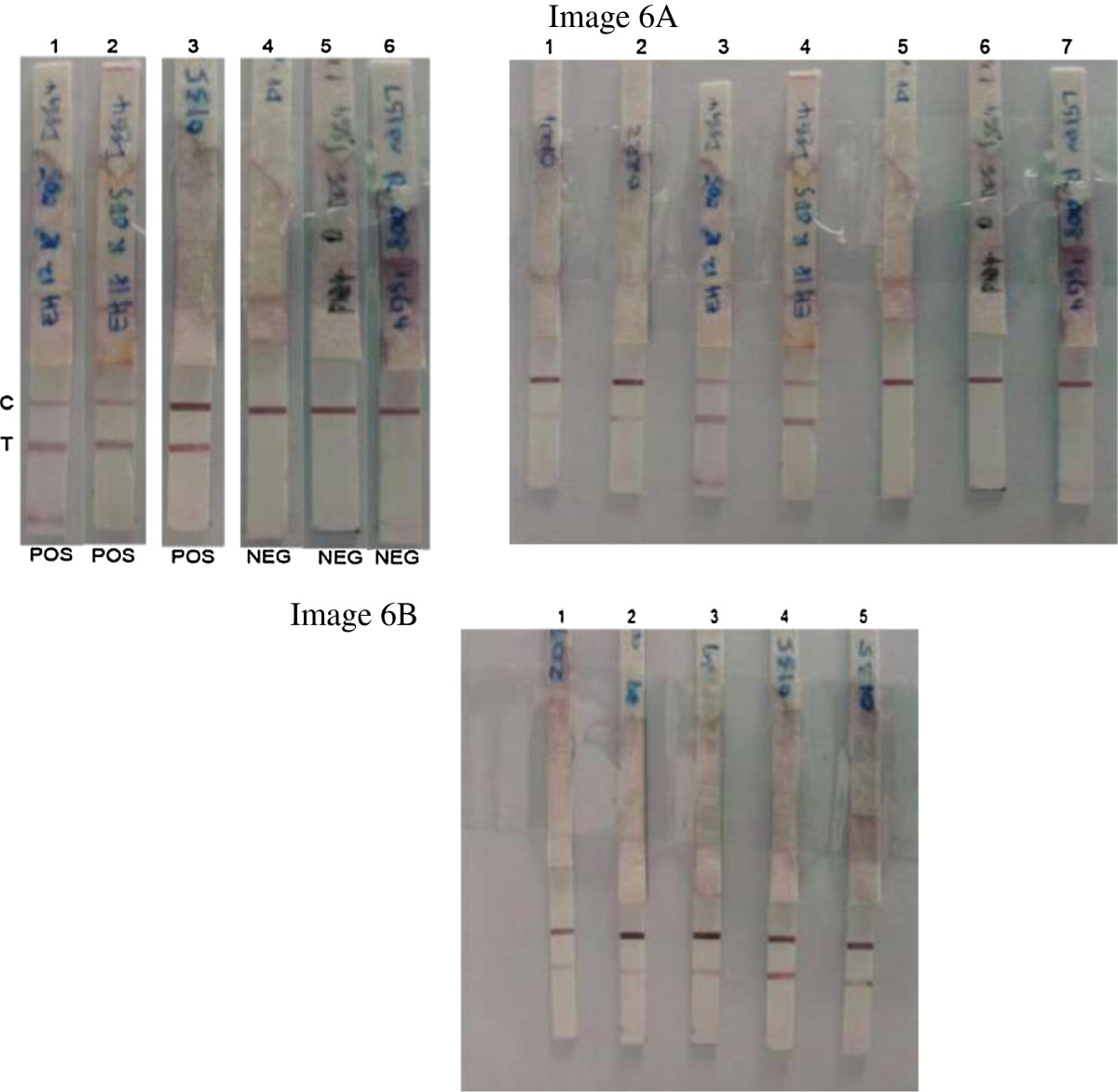
**Representative rPPDK-LFD strips tested with serum samples.** Lanes 1–3: Lateral flow test strips tested with serum samples from patients with ALA; Lane 4: strip tested with serum from healthy individual; Lanes 5–6: strips tested with serum samples from other diseases (i.e. malaria and pyogenic liver abscess); C: control line; T: test line. Figure six (Figure 3) was made by grouping elements from original Images 6**A** and 6**B** as follows: Dipstick 1 and 2 in Figure six (Figure 3) came from dipsticks 3 and 4 in Image 6**A**; dipstick 3 in Figure six (Figure 3) came from dipstick 4 in Image 6B; dipsticks 4, 5 and 6 in Figure six (Figure 3) came from dipsticks 5, 6 and 7 respectively in Image 6A.

We regret any inconvenience that this inaccuracy in the figures in the original manuscript might have caused.

## Authors’ original submitted files for images

Below are the links to the authors’ original submitted files for images. Authors’ original file for figure 1 Authors’ original file for figure 2 Authors’ original file for figure 3
