# Supplementary figures and images for: Erratum To: Production of recombinant Entamoeba histolyticapyruvate phosphate dikinase and its application in a lateral flow dipstick test for amoebic liver abscess
Source: BMC Infect Dis. 2014 Nov 10;14:533. doi: 10.1186/1471-2334-14-533 (PMC4464861; doi:10.1186/1471-2334-14-533)

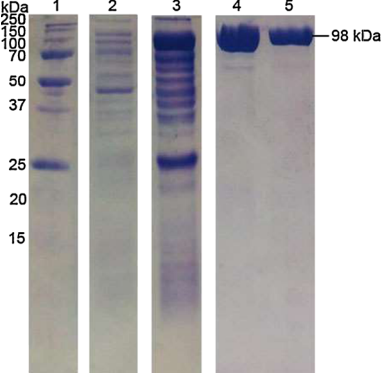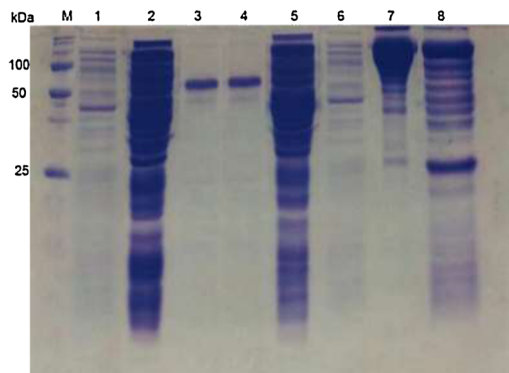

Image 1A

Image 1B

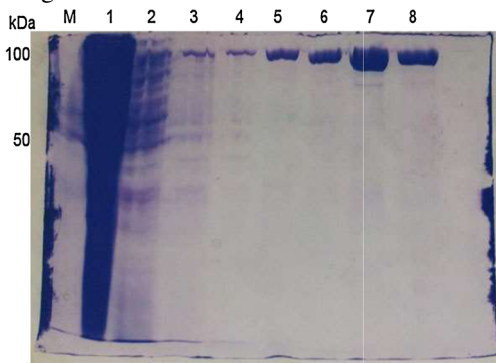

Supplement: Supplementary file 1 — Authors’ original file for figure 1 [file 12879_2014_4022_MOESM1_ESM.pdf]

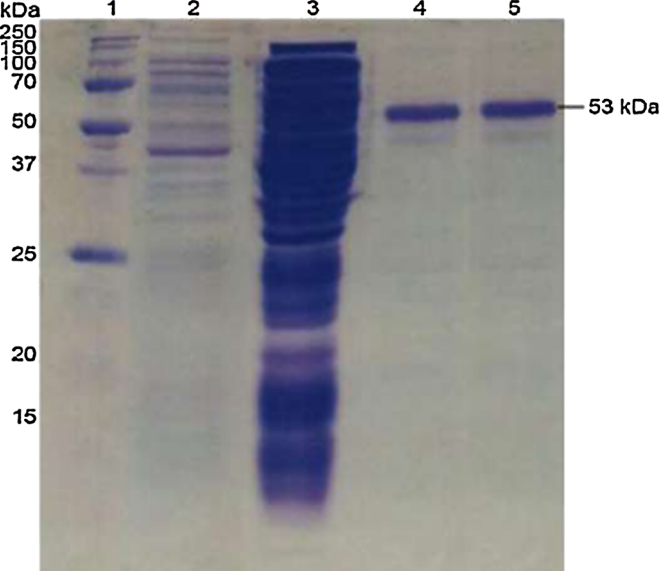

Image 1A

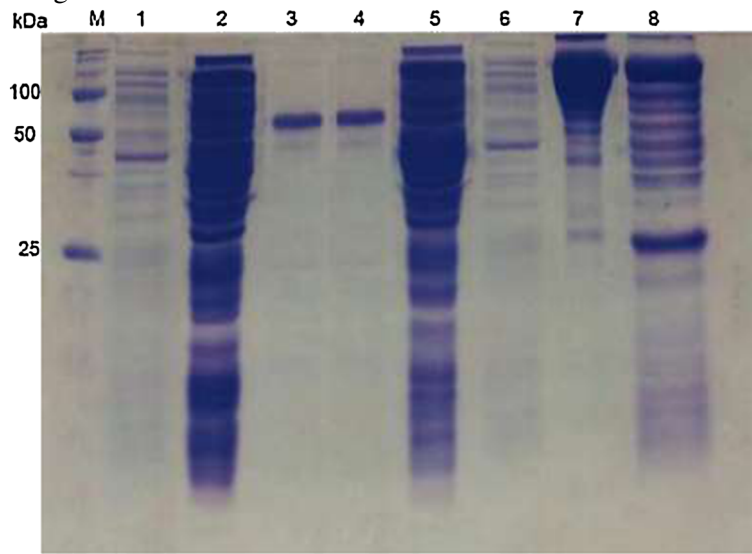

Supplement: Supplementary file 2 — Authors’ original file for figure 2 [file 12879_2014_4022_MOESM2_ESM.pdf]

Image 6A

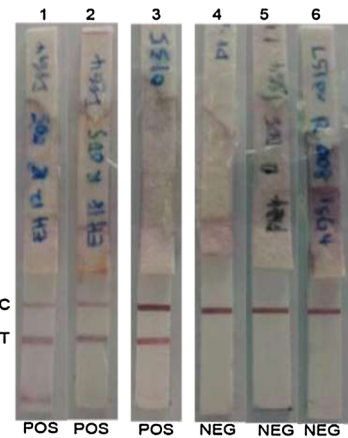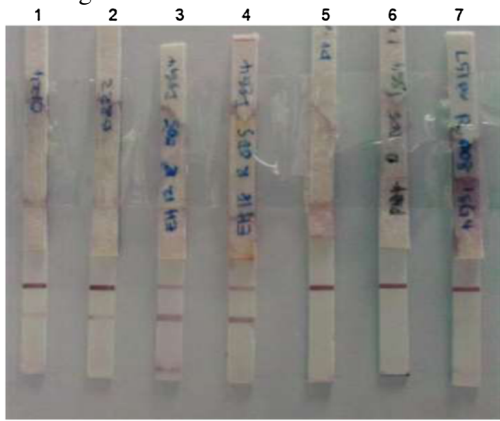

Image 6B

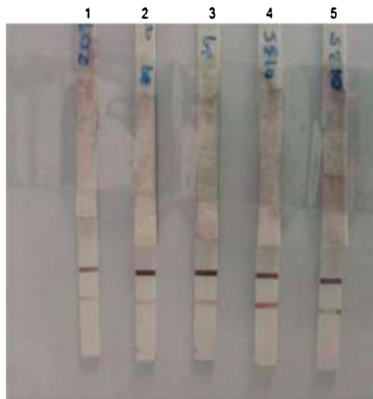

Supplement: Supplementary file 3 — Authors’ original file for figure 3 [file 12879_2014_4022_MOESM3_ESM.pdf]
